# A DFT/TD-DFT Study on the ESIPT-Type Flavonoid Derivatives with High Emission Intensity

**DOI:** 10.3390/ma15082896

**Published:** 2022-04-15

**Authors:** Xiangrui Yu, Changjiao Shang, Yunjian Cao, Jingang Cui, Chaofan Sun

**Affiliations:** College of Science, Northeast Forestry University, Harbin 150040, China; xryu@nefu.edu.cn (X.Y.); shangcj7@126.com (C.S.); yjcao@nefu.edu.cn (Y.C.)

**Keywords:** excited-state intramolecular proton transfer, photophysical property, density functional theory, intramolecular hydrogen bond, substitution effect

## Abstract

To reveal the influence of different substituents on the excited-state intramolecular proton transfer (ESIPT) process and photophysical properties of 4′-N, N-dimethylamino-3-hydroxyflavone (DMA3HF), two novel molecules (DMA3HF-CN and DMA3HF-NH_2_) were designed by introducing the classical electron-withdrawing group cyano (-CN) and electron-donating group amino (-NH_2_). The three molecules in the acetonitrile phase were systematically researched by applying the density functional theory (DFT) and time-dependent DFT (TD-DFT) methods. The excited-state hydrogen bond enhancement mechanism was confirmed, and the hydrogen bond intensity followed the decreasing order of DMA3HF-NH_2_ > DMA3HF > DMA3HF-CN, which can be explained at the electronic level by natural bond orbital, fuzzy bond order, and frontier molecular orbital analyses. Moreover, we found from the electronic spectra that the fluorescence intensity of the three molecules in keto form is relatively strong. Moreover, the calculated absorption properties indicated that introducing the electron-withdrawing group -CN could significantly improve the absorption of DMA3HF in the ultraviolet band. In summary, the introduction of an electron-donating group -NH_2_ can promote the ESIPT reaction of DMA3HF, without changing the photophysical properties, while introducing the electron-withdrawing group -CN can greatly improve the absorption of DMA3HF in the ultraviolet band, but hinders the occurrence of the ESIPT reaction.

## 1. Introduction

Excited-state intramolecular proton transfer (ESIPT), as one of the most basic modes of proton transfer in the photophysical and photochemical fields, is also a very common and important hydrogen bond dynamics behavior [1,2,3,4,5]. Since Weller first proposed the ESIPT mechanism in the middle of the last century [6], plenty of investigators have devoted themselves to experimental and theoretical studies on the ESIPT reaction, and derived various interesting research directions closely related to the ESIPT process [7,8,9,10,11,12,13,14,15,16,17,18]. The ESIPT reaction is essentially a photoisomerization behavior of molecules, which makes molecules exhibit double-fluorescence characteristics and a significant Stokes shift. Due to the large Stokes shift, there is almost no overlap between the absorption and emission of ESIPT-type molecules, which can avoid the interference of other fluorescent materials in the sample and the internal filtering effect. Moreover, on the basis of the uniquely high sensitivity of the ESIPT mechanism, it is easy to tune the fluorescence characteristics of ESIPT-type molecules. Based on these special properties, the molecules with ESIPT characteristics have been widely applied in fluorescent probes, ultraviolet filtering, chemosensors, etc. [19,20,21,22,23,24].

4′-N,N-dimethylamino-3-hydroxyflavone (DMA3HF)—an artificially synthesized fluorescent flavonoid derivative—has attracted wide attention in the fields of photophysics and chemistry due to its ESIPT characteristics, specific photophysical properties, and strong antioxidant activity [25,26,27]. Ghosh et al. [28] skillfully studied the ESIPT reaction of DMA3HF in sodium bis-ethylhexylsulfosuccinate (AOT), n-heptane, and water reverse micelle solutions by changing the ratio of water to AOT around DMA3HF encapsulated in nanovacuoles, and showed the influence of solvent polarity on the ESIPT process and photophysical properties of DMA3HF. Moreover, Das et al. [29] researched the ESIPT reaction efficiency of DMA3HF in the lyotropic liquid crystal (LLC) phase, thus exploring the hydrogen-bonding effects and polarity of LLC water molecules. Simultaneously, the effect of hydrogen bonds on the ESIPT reaction was clarified, and the slow ESIPT behavior observed experimentally in LLC was confirmed. Furukawa et al. [30] studied the effect of external electric fields on the ESIPT reaction and photophysical properties of DMA3HF in rigid polymethyl methacrylate (PMMA) films, and found that the excited-state dynamics of DMA3HF in a rigid environment are very different from those in solution. Furthermore, Ushakou et al. [31] explored the energetic characteristics of 3-hydroxyflavone (3-HF) and DMA3HF in enol and keto forms by comparing the spectroscopic properties of the two compounds in acetonitrile and ethyl acetate at different temperatures, which can provide reference for evaluating the reversibility of molecular proton transfer reactions. Nevertheless, the effect of substitution of different functional groups on the ESIPT mechanism and photophysical properties of the DMA3HF molecule is still lacking, and deserves further study.

**Figure 1:**
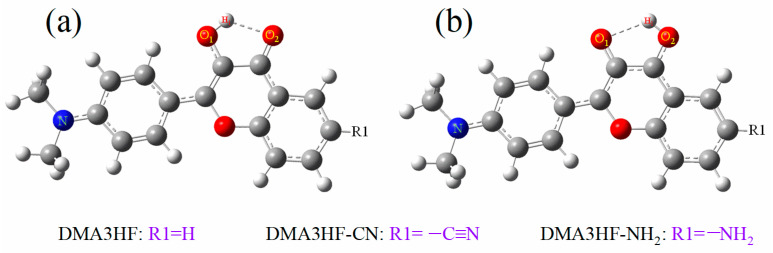
Molecular structures of DMA3HF and its derivatives in (**a**) enol and (**b**) keto forms.

For solving this issue, we designed two novel molecules (DMA3HF-CN and DMA3HF-NH_2_) by introducing the electron-withdrawing group cyano (-CN) and electron-donating group amino (-NH_2_) on the side of the proton acceptor, respectively (as can be seen in Figure 1). The -NH_2_ group as the classical strong electron-donating group with high activity, and the -CN group as the excellent hydrogen bond acceptor and strong electron-donating group, have been widely used in molecular modification. Since the stronger electron-donating group -N(CH_3_)_2_ is located at one end on the proton donor side, the introduction of functional groups at the other farthest end on the proton-acceptor side can more significantly cause the push/pull effect of -N(CH_3_)_2_ and its substituents on electrons, thereby tuning the ESIPT reaction of DMA3HF. The electron push/pull effect caused by the substitution of other carbons on the aromatic ring on the proton acceptor side is not as obvious as that of the carbon in the straight direction. Moreover, we predict that the introduction of the -CN group may weaken the electronegativity of the proton acceptor and inhibit the ESIPT process, while the introduction of the -NH_2_ group may promote the ESIPT reaction. In this work, the density functional theory (DFT) and time-dependent DFT (TD-DFT) methods were used to conduct comprehensive and detailed theoretical research on the ESIPT behavior as well as the photophysical properties of DMA3HF, DMA3HF-CN, and DMA3HF-NH_2_. The geometric parameters, corresponding infrared (IR) vibration spectra, natural bond orbital (NBO), fuzzy bond order (FBO), reduced density gradient (RDG) scatterplot, and topological parameters at the bond critical point (BCP) were obtained to explore the intensity changes of the intramolecular hydrogen bonds (IHBs), and also clarified the intensity relationships of the IHBs of DMA3HF, DMA3HF-CN, and DMA3HF-NH_2_. Moreover, the potential energy curves (PECs) were plotted to intuitively illustrate the degree of difficulty of proton transfer reaction, and the transition state (TS) structures along with the corresponding single-point energy (SPE) were obtained to calculate the exact values of the potential barriers. Furthermore, the corresponding electronic spectra of the three molecules were simulated to explore the influence of functional group substitution on the photophysical properties, and the frontier molecular orbitals (FMOs) associated with major transitions were also shown. We hope that this theoretical study on the ESIPT mechanism can provide some inspiration for the following purposeful search and synthesis of novel, high-quality, ESIPT-type fluorescent materials.

## 2. Computational Methods

In this study, the S_0_- and S_1_-state geometric configurations of the three molecules were fully optimized by the DFT and TD-DFT methods with the B3LYP functional and 6-311++G(d) basis set [32,33,34,35,36]. For keeping consistent with the experimental conditions, acetonitrile was selected as the solvent, and the polarizable continuum model (PCM) with the integral equation formalism variant (IEFPCM) was applied [37,38]. To accurately simulate the absorption and emission spectra, the fluorescence spectrum of DMA3HF in acetonitrile was calculated using seven different functionals, and the obtained results were compared with the experimental data [29], which indicated that the PBEPBE functional was the most suitable (see Table 1) [39,40,41,42,43,44]. Therefore, in this work, the absorption and fluorescence spectra for DMA3HF, DMA3HF-CN, and DMA3HF-NH_2_ were computed at the TD-DFT/PBEPBE/6-311++G(d) level. Moreover, the principal geometric parameters, IR vibration spectra, and NBO analysis were calculated based on the optimized geometric configurations [45,46,47]. Moreover, the FBO values [48], topological parameters at BCP [49], RDG scatterplots [50], IRI maps [51], and FMO maps of the three compounds were obtained using Multiwfn software (Version: 3.7, Beijing Kein Research Center for Natural Sciences, Beijing, China) and the VMD program (Version: 1.9.3, Theoretical and Computational Biophysics Group, Beckman Institute of the UIUC, Champaign-Urbana, IL, USA) [52,53]. The PECs of the three molecules in the S_0_ and S_1_ states were scanned by increasing the O_1_-H_1_ bond length with a fixed value. For the scans of the PECs, we performed restricted geometric optimizations for the three compounds. Furthermore, in order to obtain the exact values of the ESIPT reaction barriers, the corresponding TS structures of the three molecules were searched based on the quasi-Newton and synchronous transit (QST3) approach, and it was confirmed by vibration analysis that there was only one virtual mode corresponding to the proton transfer behavior [54]. We also observed the intrinsic reaction coordinate (IRC) curves to confirm that the TS structures we searched were correct [55]. All of the theoretical calculations in this work were achieved using the Gaussian 16 package (Version: Revision C.01, Gaussian, Inc., Wallingford, CT, USA) [56].

## 3. Results and Discussion

### 3.1. Optimized Geometric Structures and Infrared (IR) Vibrational Spectra Analysis

The geometric configurations of DMA3HF, DMA3HF-CN, and DMA3HF-NH_2_ in enol and keto forms in different electronic states were optimized without any restrictions via the DFT and TD-DFT methods, and the main IHB geometric parameters are presented in Table 2. The comparison of geometric parameters related to IHBs (bond lengths and angles) can show the changes in IHBs’ intensity after the photoabsorption process. As listed, for enol configurations of DMA3HF and its derivatives, the O_1_-H_1_ bond length and ∠(O_1_-O_1_⋯O_2_) bond angle were all increased by the photoexcitation process, whereas the H_1_⋯O_2_ bond lengths were shortened. Concretely speaking, the O_1_-H_1_ bonds of the three molecules were separately elongated by 0.011 Å (DMA3HF) from 0.977 Å (S_0_) to 0.988 Å (S_1_), 0.009 Å (DMA3HF-CN) from 0.977 Å (S_0_) to 0.986 Å (S_1_), and 0.010 Å (DMA3HF-NH_2_) from 0.978 Å (S_0_) to 0.988 Å (S_1_). Similarly, the H_1_⋯O_2_ bonds were reduced by 0.116 Å, 0.111 Å, and 0.113 Å, respectively, from the S_0_ to S_1_ states. Meanwhile, the angles ∠(O_1_-H_1_⋯O_2_) increased from 117.886°, 117.187°, and 118.153° in the S_0_ state to 122.179°, 121.196°, and 122.390° in the S_1_ state, respectively. These results indicate that the IHBs of DMA3HF and its derivatives are all reinforced by the photoexcitation, which can promote the occurrence of the proton transfer process.

**Figure 2:**
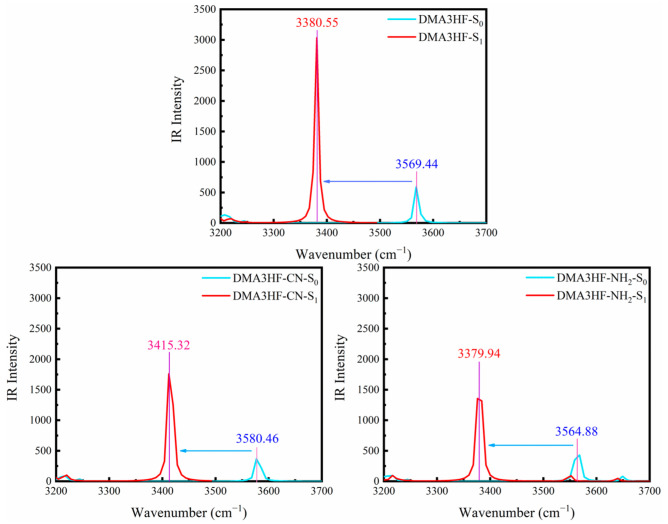
Simulated IR spectra of the three compounds in acetonitrile at the spectral region of the O_1_–H_1_ stretching band.

Monitoring the movement of stretching vibrational peaks related to IHBs in IR spectra is another available method to assess the changes in IHBs’ intensity [57,58]. Figure 2 depicts the calculated IR spectra of the three compounds at the S_0_ and S_1_ states, in the spectral range from 3200 cm^−1^ to 3700 cm^−1^, which corresponds to the region of O_1_-H_1_ stretching vibration peaks. It should be noted that the vibrational peaks of DMA3HF, DMA3HF-CN, and DMA3HF-NH_2_ all display redshifts (S_0_→S_1_) to varying degrees, and the redshift magnitude of the three compounds is 188.89 cm^−1^, 165.14 cm^−1^, and 184.94 cm^−1^, respectively. Therefore, the O_1_-H_1_ bond strength is weakened and the attraction between H_1_ and O_2_ atoms is strengthened during the photoexcitation, which can promote the occurrence of the ESIPT reaction. The above conclusions are in great agreement with the results acquired from geometric structures.

### 3.2. Natural Bond Orbital (NBO) and Fuzzy Bond Order (FBO) Analysis

On the basis of the above discussion about geometric parameters and IR spectra, we can note that the IHBs are strengthened in the S_1_ state. Hydrogen bonds, as a weak interaction, are influenced by the charge over the correlation atoms, and the redistribution of atomic charge leads to changes in the intensity of IHBs. Therefore, the NBO population analysis was performed to quantitatively research the electronegativity of proton-donor and -acceptor moieties (O_1_ and O_2_ atoms). The charge distribution on O_1_ and O_2_ atoms of DMA3HF, DMA3HF-CN, and DMA3HF-NH_2_ in the S_0_ and S_1_ states was calculated, and is summarized in Table 3. As shown, the negative charges located on the O_1_ atoms of DMA3HF, DMA3HF-CN, and DMA3HF-NH_2_ decrease from the S_0_ to the S_1_ state, while those on the O_2_ atoms increase. That is, the attraction of O_1_ atoms to hydrogen protons is attenuated, while that of O_2_ atoms to protons is improved, corresponding to the elongation of O_1_-H_1_ bonds and the shortening of H_1_⋯O_2_ bonds under the photoexcitation.

Moreover, FBO analysis was introduced to further quantitatively represent the characteristics of O_1_-H_1_ bonds and H_1_⋯O_2_ bonds. Based on the division of fuzzy atomic space, FBO can directly reflect the degree of delocalization of electrons between two atomic spaces [59]. It is well established that the larger the magnitude of the FBO, the greater the bond strength. The FBO analysis results of O_1_-H_1_ bonds and H_1_⋯O_2_ bonds in the S_0_ and S_1_ states are exhibited in Table 4. We can see that, from the S_0_ to S_1_ states, the O_1_-H_1_ bond order values of the three compounds in enol form decreased, while the H_1_⋯O_2_ bond order values all increased. This implies that the ability of O_1_ atoms to bind the protons is reduced, while that of O_2_ atoms is enhanced, in the S_1_ state. Notably, the negative charge values distributed on the proton-acceptor O_2_ atoms of the three compounds in the S_0_ and S_1_ states can both be arranged in the following order: DMA3HF-CN < DMA3HF < DMA3HF-NH_2_. Meanwhile, the order of the H_1_⋯O_2_ FBO is the same. This means that, compared with DMA3HF, the substitution of the typical electron-withdrawing group (-CN) can attract away some electrons of the O_2_ atoms, thus weakening the ability of O_2_ atoms to capture the protons. Conversely, introducing the typical electron-donating group (-NH_2_) has the opposite effect.

### 3.3. Reduced Density Gradient (RDG) Scatterplot and Topology Analysis

**Figure 3:**
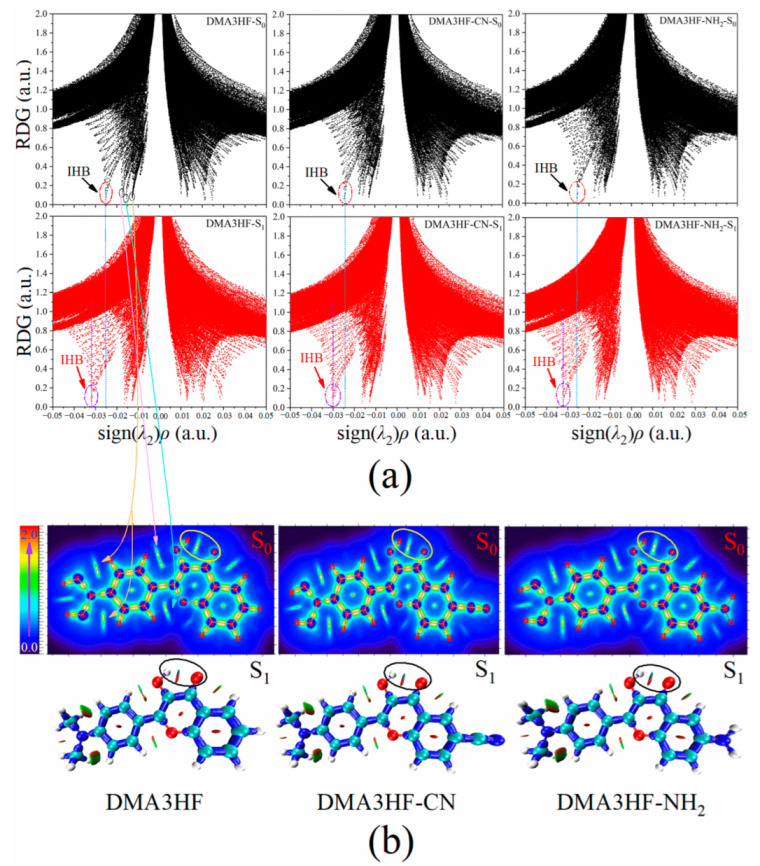
RDG scatterplots and IRI maps of DMA3HF and its two derivatives in the S_0_ and S_1_ states: (**a**) the RDG versus sign (λ_2_)*ρ* scatterplots of the three compounds; (**b**) the IRI plane color filling map in the S_0_ state and IRI isosurface map in the S_1_ state of the three compounds.

Firstly, RDG analysis was selected to visually research the changes in the IHB strength of DMA3HF, DMA3HF-NH_2_, and DMA3HF-CN. The sign (λ_2_)*ρ* function obtained by multiplying the total electron density and the sign function of the second largest eigenvalue of the Hessian matrix for electron density was projected onto the RDG isosurface, and the intensity and type of weak interactions could be clearly seen [60]. When the RDG value of the scatter point is close to 0, the point corresponds to a weak interaction. For the sign (λ_2_)*ρ* function, the *ρ-*value can represent the strength of the interaction, and the sign (λ_2_) can denote the type of interaction. When the sign (λ_2_) is equal to −1, it represents attraction, while when the sign (λ_2_) is +1, it represents repulsion. By observing the molecular structure, we can know that the IHB is the strongest weak attractive interaction in the molecule. Therefore, we can confirm that the most negative spike corresponds to the IHB. In Figure 3a, the spikes representing the IHB of each molecule are circled, and the other negative spikes representing the other weak interactions are also marked (take DMA3HF-S_0_, for example). We compared the sign (λ_2_)*ρ*-values of IHBs’ spikes in the S_0_ and S_1_ states. It was found that the values of sign (λ_2_)*ρ* were decreased by 0.0067 a.u. (DMA3HF) from −0.0253 a.u. (S_0_) to −0.0320 a.u. (S_1_), 0.0062 a.u. (DMA3HF-CN) from −0.0239 a.u. (S_0_) to −0.0304 a.u. (S_1_), and 0.0066 a.u. (DMA3HF-NH_2_) from −0.0257 a.u. (S_0_) to −0.0323 a.u. (S_1_), implying that the IHBs are indeed enhanced in the S_1_ state. In addition, we also found that the IHB strength of DMA3HF-CN was weaker than that of the other two molecules. Incidentally, all of the chemical bonds and weak interaction regions of the three compounds in the S_0_ and the S_1_ states are shown by the interaction region indicator (IRI) plane color filling map and isosurface map, respectively, which can be seen in Figure 3b.

However, the sign (λ_2_)*ρ*-values of DMA3HF and DMA3HF-NH_2_ are very close, and cannot be distinguished only by the spikes in the RDG maps. Hence, we selected the atoms in molecules (AIM) theory to obtain the topological parameters at the bond critical points (BCPs) of the IHBs, and directly calculated the hydrogen bond energy (E_HB_) of the three compounds in the S_0_ and S_1_ states using empirical formulae [61,62]. The relevant parameters are listed in Table 5. Therein, the Laplacian of electron density ∇^2^*ρ*(r) values are positive, representing closed-shell interactions (corresponding to IHBs in this paper). In addition, the corresponding hydrogen bond was considered to be strong when the *ρ*(r) value at BCP was larger than 0.03 a.u., and the larger the value of *ρ*(r), the stronger the IHB. All of the relevant parameters indicate that the IHBs of the three molecules are strengthened in the S_1_ state. It is worth noting that the order of E_HB_ for the three compounds is DMA3HF-CN < DMA3HF < DMA3HF-NH_2_, whether in the S_0_ or S_1_ state. This result directly illustrates that the substitution of the electron-donating group -NH_2_ on the proton-acceptor side can promote the ESIPT process; however, the substitution of the electron-withdrawing group -CN is able to inhibit the ESIPT behavior. The RDG and topology analysis provide proof for our previous conclusions acquired from FBO and NBO analyses.

### 3.4. Potential Energy Curves (PECs)

**Figure 4:**
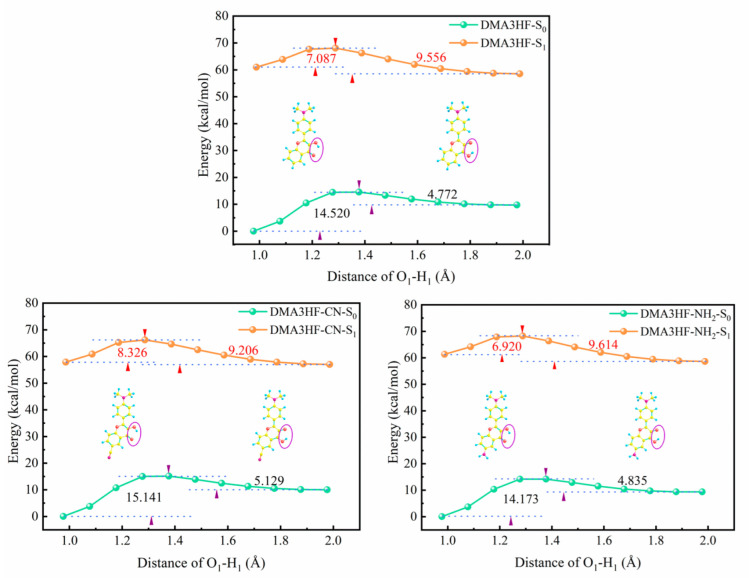
Scanned PECs of the three molecules in the S_0_ and S_1_ states.

In order to further intuitively illustrate the degree of difficulty of proton transfer in the S_0_ and S_1_ states for DMA3HF, DMA3HF-CN, and DMA3HF-NH_2_, we scanned the PECs of the three molecules via lengthening the O_1_-H_1_ bonds from 1.0 Å to 2.0 Å, at a fixed step of 0.1 Å, and allowing all other degrees of freedom to relax freely towards the minimum energy [63,64], as shown in Figure 4. It can be seen that, for the three investigated molecules, the energy barriers for the forward proton transfer process in the S_0_ state were significantly larger than those in the S_1_ state, implying that the proton transfer behaviors are more likely to occur in the excited state. Moreover, the order of barriers for the three compounds in the S_0_ and S_1_ states are both DMA3HF-NH_2_ < DMA3HF < DMA3HF-CN, implying that the substitution of the -CN group at the proton-acceptor side impedes proton transfer, while the substitution of the -NH_2_ group promotes proton transfer, which is consistent with the results of NBO, FBO, RDG, and topology analyses.

**Figure 5:**
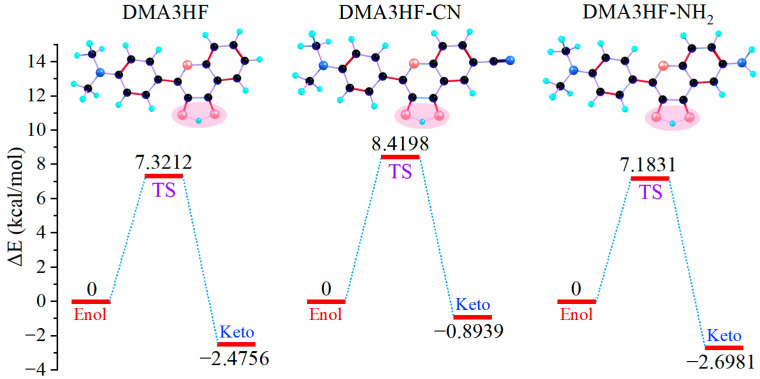
ESIPT reaction energy profiles for DMA3HF and its two derivatives.

**Figure 6:**
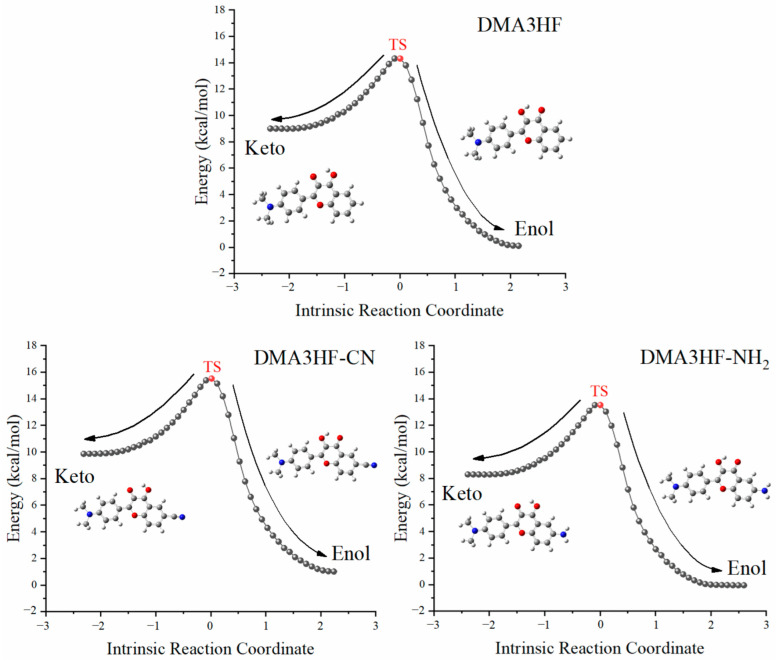
IRC curves scanned based on the TS structures of the three molecules.

Nevertheless, due to the limitation of scanning step length, the above PECs cannot accurately describe the reaction path of ESIPT. Therefore, it is necessary to search the transition state (TS) structures of molecules during proton transfer, and to accurately calculate the corresponding single-point energy (SPE). The SPE of all of the stable structures in the S_1_ state was calculated, as can be seen in Figure 5. As shown, the energy barriers for the ESIPT process were 7.1831 kcal/mol (for DMA3HF-NH_2_), 7.3212 kcal/mol (for DMA3HF), and 8.4198 kcal/mol (for DMA3HF-CN). This result confirms once again that the substitution of the -CN group on the proton-acceptor side hindered the ESIPT reaction, and the substitution of the -NH_2_ group would have the opposite effect. Furthermore, we drew the intrinsic reaction coordinate (IRC) curves based on the TS structures of the three molecules. As shown in Figure 6, the two ends of the IRC curves correspond to the enol and keto forms of the molecules, respectively, proving that the TS structures of the ESIPT reaction for which we searched were correct.

### 3.5. Electronic Spectra and Frontier Molecular Orbitals (FMOs)

**Figure 7:**
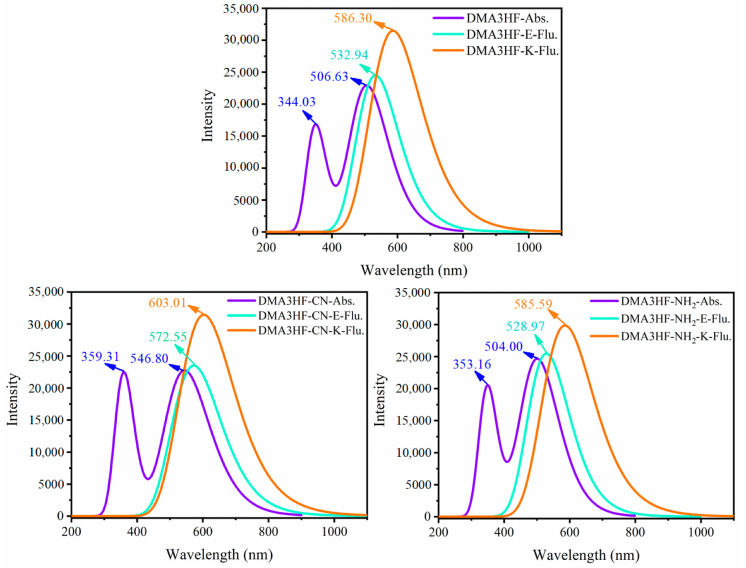
Simulated absorption and fluorescence spectra of the three molecules in the enol and keto forms in acetonitrile.

**Figure 8:**
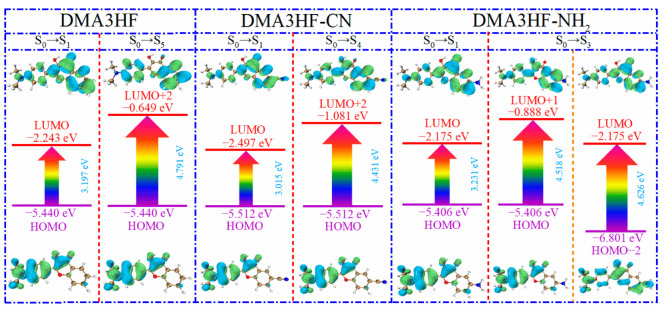
Frontier molecular orbitals and energy gaps of the three compounds.

In this section, we explored the effects of two classical types of functional groups (-CN and -NH_2_) on the photophysical properties of DMA3HF. On the basis of the optimized ground- and excited-stated structures, the absorption and emission spectra of DMA3HF, DMA3HF-CN, and DMA3HF-NH_2_ were simulated at the IEFPCM/TD-DFT/PBEPBE/6-311++G(d) level, and are displayed in Figure 7. Moreover, the transition properties (e.g., transition composition and oscillator strengths *f*) associated with the six low-lying absorption transitions (S_1_–S_6_) in acetonitrile are summarized in Table 6, and the fluorescence properties are listed in Table 7. As shown in Figure 7, the calculated fluorescence peaks of DMA3HF in the enol and keto forms are separately located at 532.94 nm and 586.30 nm, corresponding with the experimental values of 510 nm and 570 nm [29], and further indicating that the selected theoretical level is suitable for simulating the electronic spectra of DMA3HF, DMA3HF-CN, and DMA3HF-NH_2_. This also indirectly proves that the geometric structures optimized by the B3LYP functional are accurate. Notably, all three molecules possess obvious double-absorption peaks. Compared with the absorption spectra of DMA3HF, introducing the electron-donating group (-NH_2_) induced the absorption intensity of the dual peaks to increase to varying degrees, and the absorption peak located in the long-wavelength band exhibits a tiny blueshift of 2.63 nm. Moreover, the absorption peak of DMA3HF-CN in the long-wavelength band shows a redshift of 40.17 nm compared with that of DMA3HF, and the intensity of the absorption peak in the short-wavelength band increased obviously—even as high as the absorption peak in the long-wavelength band. As listed in Table 6, the absorption peaks of the three compounds in the long-wavelength band were ascribed to the S_0_→S_1_ transition, which was generated by the electronic transition from the highest occupied molecular orbital (HOMO) to the lowest unoccupied molecular orbital (LUMO). Furthermore, the absorption peaks of DMA3HF, DMA3HF-CN, and DMA3HF-NH_2_ in the short-wavelength band correspond to the S_0_→S_5_ (HOMO→LUMO+2), S_0_→S_4_ (HOMO→LUMO+2), and S_0_→S_3_ transitions (HOMO-2→LUMO and HOMO→LUMO+1), respectively. Based on the transition properties of the three molecules, the corresponding FMO and energy gap diagrams are plotted in Figure 8. The occurrence of intramolecular charge transfer (ICT) behavior under the photoexcitation can be visually observed [65,66]. Notably, for the transition (S_0_→S_1_), the electronic cloud density distributed over the O_1_ atoms of the three compounds decreased, while that over the O_2_ atoms increased. That is, the ability of O_1_ atoms to attract protons was reduced, while that of O_2_ atoms is enhanced, which can advance the occurrence of the ESIPT reaction. Moreover, the peak shift phenomenon in the absorption spectra can be deduced from the energy gaps of the corresponding orbitals. It also can be seen that all of the absorption peaks of the three molecules originated from the π→π* transition of electrons.

Furthermore, we can see from Figure 7 and Table 7 that, compared with DMA3HF, the dual-fluorescence signals of DMA3HF-CN redshifted by 39.61 nm (enol form) and 16.71 nm (keto form). Similarly, the fluorescence peak of DMA3HF-NH_2_ in enol form blueshifted by 3.97 nm. It is noteworthy that the fluorescence intensities of the three molecules in the keto form were very strong. Moreover, the fluorescence peaks of the three molecules in keto form exhibited Stokes shifts of 79.67 nm (DMA3HF), 56.21 nm (DMA3HF-CN), and 81.59 nm (DMA3HF-NH_2_). The above results indicate that the substitution of the -CN group causes obvious redshift phenomena in the absorption and fluorescence spectra, and significantly enhances the absorption in the short-wavelength (ultraviolet) band. However, the substitution of the -NH_2_ group had no obvious effect on the photophysical properties of DMA3HF.

## 4. Conclusions

In this work, the influences of the electron-donating group -NH_2_ and electron-withdrawing group -CN on the ESIPT mechanism and photophysical properties of DMA3HF were comprehensively studied via DFT/TD-DFT methods. Based on the results obtained from the relevant geometric parameters, IR spectra, NBO charge population, FBO, RDG isosurface, and topology analysis, the excited-stated IHB strengthening mechanisms of DMA3HF and its two derivatives were confirmed. Moreover, according to the calculated PECs and TS structures corresponding to the ESIPT process, it was found that the substitution of -NH_2_ on the proton-acceptor side can promote the ESIPT process, while the substitution of -CN shows the opposite effect. In addition, from the simulated electronic spectra, it can be seen that DMA3HF and its two derivatives show strong fluorescence in the keto configuration compared with that in the enol configuration, and the introduction of -CN can greatly enhance the absorption intensity of DMA3HF in the ultraviolet band. In conclusion, the introduction of electron-donating and electron-withdrawing groups can regulate the ESIPT process of flavonoids and, thus, affect their optical properties. This theoretical investigation can provide valuable guidance in the experimental design and synthesis of efficient ESIPT-based fluorescence materials.

## Figures and Tables

**Table 1:** Calculated fluorescence peaks (nm) of DMA3HF in acetonitrile, obtained via the TD-DFT method with seven different functionals.

	PBEPBE	B3PW91	Cam-B3LYP	B3LYP	M062x	mPW1PW91	ωB97XD	Exp ^a^
λ_flu_ 1	532.94	456.48	396.43	458.03	396.64	441.74	388.21	510
λ_flu_ 2	586.30	545.90	520.40	547.40	513.09	537.39	518.42	570

^a^ Maximum fluorescence peaks in the experiment.

**Table 2:** Calculated bond lengths (Å) and bond angles (°) related to the IHBs of DMA3HF and its derivatives in enol and keto forms in the S_0_ and S_1_ states, respectively.

	State	O_1_-H_1_	H_1_-O_2_	∠(O_1_-H_1_⋯O_2_)
DMA3HF-enol	S_0_	0.977	2.025	117.886
	S_1_	0.988	1.909	122.179
DMA3HF-keto	S_0_	1.936	0.988	120.425
	S_1_	2.009	0.981	117.761
DMA3HF-CN-enol	S_0_	0.977	2.044	117.187
	S_1_	0.986	1.933	121.196
DMA3HF-CN-keto	S_0_	1.960	0.987	119.440
	S_1_	2.018	0.981	117.213
DMA3HF-NH_2_-enol	S_0_	0.978	2.019	118.153
	S_1_	0.988	1.906	122.390
DMA3HF-NH_2_-keto	S_0_	1.934	0.988	120.556
	S_1_	2.004	0.981	118.011

**Table 3:** Calculated distribution of NBO charges (a.u.) on the O_1_ and O_2_ atoms of DMA3HF, DMA3HF-CN, and DMA3HF-NH_2_ in the S_0_ and S_1_ states.

	DMA3HF			DMA3HF-CN			DMA3HF-NH_2_		
State/Δ	S_0_	S_1_	Δ	S_0_	S_1_	Δ	S_0_	S_1_	Δ
O_1_	−0.6917	−0.6580	−0.0337	−0.6860	−0.6540	−0.0320	−0.6947	−0.6639	−0.0308
O_2_	−0.6948	−0.7646	+0.0698	−0.6819	−0.7413	+0.0594	−0.7024	−0.7741	+0.0717

Δ: Difference in NBO charges between the S_0_ and S_1_ states; positive values represent increases and negative values represent decreases (S_0_→S_1_).

**Table 4:** Obtained fuzzy bond order related to the ESIPT process.

	State	FBO (O_1_-H_1_)	FBO (H_1_⋯O_2_)
DMA3HF-enol	S_0_	0.75626	0.06129
	S_1_	0.72953	0.07995
DMA3HF-keto	S_0_	0.08263	0.72416
	S_1_	0.07044	0.74092
DMA3HF-CN-enol	S_0_	0.75760	0.05773
	S_1_	0.73284	0.07482
DMA3HF-CN-keto	S_0_	0.07747	0.72767
	S_1_	0.06834	0.73979
DMA3HF-NH_2_-enol	S_0_	0.75591	0.06242
	S_1_	0.72957	0.08095
DMA3HF-NH_2_-keto	S_0_	0.08309	0.72530
	S_1_	0.07136	0.74132

**Table 5:** Calculated topological parameters at BCPs related to the IHBs of the three molecules in enol and keto forms in the S_0_ and S_1_ states.

	*ρ*(r) ^α^	∇^2^*ρ*(r) ^β^	V(r) ^γ^	G(r) ^δ^	H(r) ^ε^	ELF ^ζ^	E_HB_^η^
DMA3HF-enol-S_0_	0.0253	0.1026	−0.0204	0.0230	0.0027	0.0689	−4.9016
DMA3HF-enol-S_1_	0.0320	0.1209	−0.0270	0.0286	0.0016	0.0950	−6.3963
DMA3HF-keto-S_0_	0.0310	0.1133	−0.0254	0.0268	0.0015	0.0964	−6.1732
DMA3HF-keto-S_1_	0.0267	0.1009	−0.0212	0.0232	0.0020	0.0805	−5.2139
DMA3HF-CN-enol-S_0_	0.0239	0.1241	−0.0222	0.0266	0.0044	0.0439	−4.5893
DMA3HF-CN-enol-S_1_	0.0304	0.1168	−0.0253	0.0272	0.0020	0.0887	−6.0393
DMA3HF-CN-keto-S_0_	0.0294	0.1095	−0.0238	0.0256	0.0018	0.0899	−5.8163
DMA3HF-CN-keto-S_1_	0.0262	0.1000	−0.0207	0.0229	0.0022	0.0781	−5.1024
DMA3HF-NH_2_-enol-S_0_	0.0257	0.1035	−0.0207	0.0233	0.0026	0.0704	−4.9909
DMA3HF-NH_2_-enol-S_1_	0.0323	0.1215	−0.0272	0.0288	0.0016	0.0959	−6.4632
DMA3HF-NH_2_-keto-S_0_	0.0311	0.1137	−0.0255	0.0270	0.0015	0.0969	−6.1955
DMA3HF-NH_2_-keto-S_1_	0.0270	0.1017	−0.0214	0.0234	0.0020	0.0817	−5.2809

α: Density of all electrons (a.u.); β: Laplacian of electron density (a.u.); γ: potential energy density (a.u.); δ: Lagrangian kinetic energy (a.u.); ε: energy density (a.u.); ζ: electron localization function (a.u.); η: hydrogen bond energy (kcal/mol), E_HB_ = –223.08*ρ*(r) + 0.7423.

**Table 6:** Calculated transition properties of the three compounds in acetonitrile.

	State	*λ*_abs_ (nm)	Contribution MO ^a^	Strength *f*
DMA3HF	S_1_	506.63	(68.325%) H→L	0.5655
	S_2_	369.48	(56.740%) H→L + 1 (34.285%) H→L + 2	0.1037
	S_3_	366.37	(67.241%) H-1→L	0.0431
	S_4_	344.55	(70.680%) H-2→L	0.0000
	S_5_	344.03	(55.193%) H→L + 2	0.2958
	S_6_	329.47	(45.859%) H-3→L (46.421%) H→L + 3	0.0086
DMA3HF-CN	S_1_	546.80	(67.535%) H→L	0.5329
	S_2_	504.39	(69.415%) H→L + 1	0.0359
	S_3_	383.18	(65.797%) H-1→L	0.0241
	S_4_	359.31	(63.652%) H→L + 2	0.5336
	S_5_	355.10	(70.573%) H-2→L	0.0000
	S_6_	343.13	(66.814%) H-3→L	0.0040
DMA3HF-NH_2_	S_1_	504.00	(68.324%) H→L	0.5836
	S_2_	445.18	(67.881%) H-1→L	0.0474
	S_3_	353.16	(47.579%) H-2→L (46.050%) H→L + 1	0.3820
	S_4_	343.13	(69.751%) H-3→L	0.0051
	S_5_	339.41	(45.346%) H-2→L (45.640%) H→L + 1	0.1166
	S_6_	336.45	(58.989%) H→L + 2 (31.026%) H→L + 3	0.0147

a: Molecular Orbitals; H: the highest occupied molecular orbital (HOMO); L: the lowest unoccupied molecular orbital (LUMO).

**Table 7:** Calculated fluorescence properties of the three molecules in the enol and keto forms in acetonitrile.

	State	E_flu_ (eV)	λ_flu_ (nm)	Contribution MO ^a^	Strength *f*
DMA3HF-enol	S_1_	2.3264	532.94	H→L (68.670%)	0.6049
DMA3HF-keto	S_1_	2.1147	586.30	H→L (71.559%)	0.7774
DMA3HF-CN-enol	S_1_	2.1655	572.55	H→L (68.886%)	0.5805
DMA3HF-CN-keto	S_1_	2.0561	603.01	H→L (70.403%)	0.7765
DMA3HF-NH_2_-enol	S_1_	2.3439	528.97	H→L (68.663%)	0.6291
DMA3HF-NH_2_-keto	S_1_	2.1173	585.59	H→L (71.017%)	0.7374

a: Molecular Orbitals; H: the highest occupied molecular orbital (HOMO); L: the lowest unoccupied molecular orbital (LUMO).

## Data Availability

The data presented in this study are available upon request from the corresponding author.
